# Genetic and phenotypic evaluation of milk production in Khuzestani buffalo under intensive farming conditions

**DOI:** 10.1016/j.vas.2026.100589

**Published:** 2026-01-30

**Authors:** Kobra Karimi, Mohammad Taghi Beigi Nassiri, Mahmoud Amiri Roudbar, Alireza Jolazadeh

**Affiliations:** a Department of Animal Science, Khuzestan Agricultural Sciences and Natural Resources University, Ahvaz, Iran; b Department of Animal Science, Safiabad-Dezful Agricultural and Natural Resources Research and Education Center, Agricultural Research, Education & Extension Organization (AREEO), Dezful, Iran

**Keywords:** Khuzestani buffalo, Milk yield, Heritability, Genetic trend

## Abstract

Understanding the productive potential of Khuzestani buffaloes is essential for improving dairy efficiency and supporting sustainable livelihoods in regions where this indigenous breed is central to agriculture. This study evaluated milk yield and genetic parameters of Khuzestani buffaloes managed under intensive farming conditions. A total of 33,521 test-day milk (TDM) records from 106 buffaloes collected between 2021 and 2025 were analyzed using polynomial mixed models adjusted for biological, environmental, and management effects. The 270-day milk yield (270DMY) was estimated, and variance components were derived using a Bayesian linear mixed model with Gibbs sampling. Average daily milk yield was 4.82 kg, with peak production observed between 50 and 75 days in milk and higher yields in animals milked without calves. The mean 270DMY was 1065 ± 355.8 kg, with a significant annual increase of 66.75 kg, indicating steady herd-level improvements. Genetic analysis revealed a moderate heritability for 270DMY (h² = 0.433) and a positive genetic trend of ∼28.5 kg/year in estimated breeding values, confirming measurable progress through selective breeding. As a first-step report focused on milk yield, we demonstrate substantial untapped genetic potential in Khuzestani buffalo and quantify key productivity drivers—milking type, season, parity, age at calving, and temperature. Overall, the findings provide the first comprehensive genetic evaluation of this indigenous population under standardized intensive farming with complete, verified management and pedigree records, establishing a framework for data-driven selection to enhance milk yield and resilience.

## Highlights

• First genetic assessment of milk yield in Khuzestanian buffaloes under extensive systems.
• Genetic and phenotypic trends demonstrate measurable progress in buffalo milk productivity.
• Environmental and management factors significantly influenced yield and lactation curve in buffaloes.
• Results provide a framework for sustainable breeding strategies in Iranian buffaloes.

## Introduction

Water buffalo (*Bubalus bubalis*) are a vital component of global dairy systems, contributing approximately 15.4 % of total milk production in 2022—second only to cattle, which account for about 81 % (Rashid et al., 2024). Globally, there are over 200 million water buffaloes—over 98 % of which reside in Asia—indicating their prominence in warmer regions (FAOSTAT, 2025; Rashid et al., 2024). This species produces milk that is notably richer in fat, protein, total solids, minerals, and energy compared to cow milk, making it highly suitable for processing into value-added dairy products like cheese, yogurt, ice cream, and other conventional dairy products (Emakpor et al., 2024). In 2023, global buffalo milk production surpassed 150 million tonnes, with India and Pakistan leading the sector. In the same year, Iran ranked among the top ten global producers, with an estimated annual output of approximately 128,000 tonnes from its buffalo population (FAOSTAT, 2025). The majority of this production originates from the Khuzestani buffalo, a population of around 97,000 heads located primarily in Khuzestan province, which constitutes over half of Iran’s total buffalo herd (circa 180,000 heads) and plays a pivotal role in national buffalo dairy supply.

Situated in southwestern Iran, the Khuzestani buffalo is one of three principal indigenous ecotypes—alongside Azeri and Mazandrani—exhibiting unique adaptation to the arid, high-temperature environment of Khuzestan province (Rafiepour et al., 2021; Safari et al., 2018). These buffaloes are among the largest in the world, weighing between 600 and 800 kg, with a typical lactation period of 210–250 days (Rafiepour et al., 2021). Reported estimates of milk production for this breed vary across studies, ranging from 1865 to over 2300 kg for the third lactation (Borghese, 2005; Madad et al., 2013b). Such variation, together with evidence of moderate heritability for milk yield and composition traits in Khuzestani buffaloes (Madad et al., 2013b), highlights the potential for genetic improvement through selective breeding. Studies indicate that selection based on key lactation traits can yield sustained gains in productivity, making breeding programs a crucial strategy for enhancing milk yield and overall dairy efficiency in this important indigenous population (Madad et al., 2013b; Neysi et al., 2023). Recognizing the strategic importance of the buffalo (*B. bubalis*) as the principal indigenous livestock species of Khuzestan Province, and the need for precise assessment of its productive potential, the Iranian Ministry of Agriculture Jihad established the first intensive management research nucleus herd for breeding and husbandry of Khuzestani buffaloes in 1994. This initiative was implemented at the Safiabad Agricultural and Natural Resources Research Center, Dezful, with the objective of facilitating systematic research and genetic improvement programs for the species. The foundation of this herd was based on the collection of 80 buffaloes from across Khuzestan Province, thereby making it a representative and reliable population for studying the performance of this ecotype. Over the years, this herd has generated highly valuable data on a range of economically important traits—particularly milk production—collected under controlled management conditions, thereby ensuring high accuracy and reliability of the recorded information.

We selected milk yield as the primary endpoint for this first report because it is the main bottleneck for adopting intensive management and for building a practical selection program (Shao et al., 2021). Establishing genetic parameters and trends for yield provides the foundation for herd improvement and for investment decisions. At the same time, composition traits (fat, protein, lactose, total solids) and udder health (somatic cell count) are central to product value and welfare. These traits are currently under analysis in parallel projects and will be reported separately in multi-trait genetic evaluation studies. The present study provides the first population-level genetic evaluation of Khuzestani buffaloes maintained under standardized intensive management and controlled recording conditions, using verified pedigree and environmental data. Unlike earlier studies based on traditional farm records, this work leverages a research-nucleus herd to generate reliable estimates and to quantify management factors such as milking type and temperature effects on yield. Although comprehensive breeding objectives for buffalo should integrate milk yield with other economic traits such as milk composition, fertility, and adaptability, this study focuses on milk yield as the primary driver of productivity under intensive management. Establishing this baseline is essential to demonstrate how Khuzestani buffalo perform and respond to selection in real breeding conditions before expanding analyses to additional traits.

## Material and methods

### Data

Test-day milk (TDM) production data were collected from October 2021 to February 2025 from a breeding herd maintained at the Department of Animal Science, Safiabad-Dezful Agricultural and Natural Resources Research and Education Center, Agricultural Research, Education & Extension Organization (AREEO), Dezful, Iran. The pedigree file contained 1125 buffaloes, and its structure and inbreeding statistics were computed using CFC software (Sargolzaei et al., 2006), with a full summary provided in Supplementary Table S1. At the start, the dataset comprises a total of 33,521 records from 106 female Khuzestani buffaloes. Milking was conducted twice daily, once in the morning and once in the afternoon. The total daily milk yield for each buffalo was calculated by summing the amounts from both milking sessions each day. Milk ejection was induced using two methods: 1) Induction by the presence of the calf during milking for calvings before April 2023, and 2) Induction by oxytocin injection and manual prestimulation for calvings after April 2023. In the latter, milk let-down was supported with oxytocin (Rooran Darou Pharmaceutical Co., Iran; 10 IU/mL) administered intramuscularly 1–2 min before milking: 2 mL (20 IU) daily in week 1 postpartum, tapered to 1 mL (10 IU) in week 2 and 0.5 mL (5 IU) in week 3, then discontinued if let-down was adequate; thereafter, warm-water manual pre-stimulation was used. Buffaloes that produced an average of <0.5 kg of milk per day for two weeks before reaching 270 days of milking were dried off to prepare for the next calving.

Records of TDM collected during instances of animal disease were excluded from the dataset (*n* = 1875). Additionally, records for milking days exceeding 270 days were excluded (*n* = 199). Records for calving numbers exceeding seven were removed due to an insufficient sample size (*n* = 1871). Furthermore, records for five buffaloes that had fewer than five entries for a specific calving number were excluded from the dataset (*n* = 12). After this rigorous quality control process, a total of 29,564 records from 97 buffaloes remained for further analysis.

### Fixed and covariate effects on the test-day milk production

TDM was adjusted for a range of biological, management, and environmental factors to account for sources of variation in yield. These included: day of milking (DM; 1–270 days in milk), calving number (CN; 1–7), age at calving (AC; 2.7–12 years), milking type (MT; with or without calf presence for milk letdown), weight at calving (WC; 346–764 kg), average temperature on the milking day (AT; 8.5–43.1 °C), days pregnant (DP; 0–244 days), and season of the year (SN; spring, summer, fall, or winter). The DP was calculated based on the next calving date, assuming that the average gestation period in Khuzestani buffaloes is 310 days. For milking records without information for DP (*n* = 10,623), the DP was predicted using all data from the pedigree. Based on pedigree we had 385 available records for fitting the days open (DO) in the herd. First we fit a full model with CN, MT, SN and AC effects as a backbone model. This full model was used for model selection based on BIC using stepwise function in R (R Core Team, 2020). The optimal model based on BIC for predicting DP was:

$$DO_{i}=\beta_{0}+\beta_{1}MT_{i}+\beta_{2}AC_{i}+\beta_{3}SN_{i}+\varepsilon_{i}$$

where, $\beta_{0}$,$\;\beta_{1}$,$\;\beta_{2}$,$\;\beta_{3}$ are the coefficients to be estimated, and $\varepsilon_{i}$ is the error term for the *i*th buffalo, assumed to be normally distributed, $\varepsilon_{i}∼N\left(0,\sigma_{\varepsilon }^{2}\right)$, where $\sigma_{\varepsilon }^{2}$ represents the residual variance.

### Statistical model

#### Polynomial degree optimization

The initial model to predict TDM was specified as follows:

TDM=poly(DM,2)*MT+SE+WC+CN+AC+PD+AT+τ

where poly(•, k) denotes a polynomial of degree k, and τ is the residual error, assumed to be normally distributed, $\tau ∼N(0,\sigma_{\varepsilon }^{2})$, where $\sigma_{\tau }^{2}$ represents the residual variance. To identify the optimal polynomial degrees for each continuous predictor variable, the poly for DM, WC, AC, PD, and AT were systematically varied up to maximum values of 9, 5, 5, 5, and 3, respectively, and the Bayesian Information Criterion (BIC) was calculated for each. The model with the lowest BIC was selected as the final model to ensure the best balance between goodness of fit and model complexity. The optimal polynomial degrees were identified as 9 for DM, 5 for AC, 1 for PD, 5 for WC, and 2 for AT.

#### Effect selection and adjustment

Following the identification of the optimal polynomial degrees for the covariates, the refined base model was employed for further enhancement. The stepwise function in R, guided by the BIC, was used to meticulously select the effects for the final TDM model. After selecting the fixed effects, a random animal effect was incorporated into the linear mixed model to account for the random effect of individual animals. Instead of using the additive relationship matrix, the identity matrix (I) was employed for the random effect, assuming uniform variance components across all animals. The final model, incorporating the random animal effect and adjusting for all possible fixed and covariate effects, was as:

y=Xβ+Zu+ω

where, X is the design matrix for fixed effects, β is the vector of fixed effect coefficients, Z represents the design matrix for the random animal effect, ***u*** denotes the vector of random animal effect, and ω is the vector of residual error term. The random animal effects are assumed to follow a normal distribution as $u∼N(0,\;I\sigma_{u}^{2})$, where $\sigma_{u}^{2}$ represents the variance component for the random animal effect, and the I is used for the random effect variance structure. ω was assumed to be independent and identically distributed following $e∼N(0,\;I\sigma_{\omega }^{2})$, where $\sigma_{\omega }^{2}$ is the residual variance component. The TDM was fitted with a random animal effect under identity covariance to soak up between-animal heterogeneity (additive + non-additive genetics and permanent environment) without decomposing sources of variance. This stage was used solely to obtain unbiased, precise fixed-effect estimates and model-adjusted TDM predictions. Adjusted 270-day milk yield (270DMY) was then derived by aggregating adjusted TDM across the lactation window.

To visualize the interaction between DM and MT, A linear regression model was fitted to calculate residuals using the best model, but excluding DM and interaction between DM and MT. The entire modeling procedure was performed using R (R Core Team, 2020). We estimated adjusted (marginal) means for categorical factors (MT, SE, and CN) and average marginal effects for continuous covariates (DM, WC, AC, PD, and AT) from the best-fitting linear mixed model. Adjusted means and pairwise contrasts were obtained with the emmeans package in R (Lenth, 2020), averaging over the empirical distribution of all other covariates in the model. For time/temperature terms, we retained raw polynomial parameterizations to maintain interpretability on the original scales.

#### Estimating the 270-Day milk yield

To estimate the 270DMY from TDM information, the best mixed random model was employed. The fixed-effect values were set to reflect herd-level averages: CN = 3, and the corresponding averages for AC = 6 years and WC = 517 kg, which were calculated conditional on this average CN. In addition, the mean AT = 26 °C, MT = “without calf”, and SN = “summer” were applied. Using these representative values ensures that the predicted 270DMY reflects more realistic production conditions for this herd. This model ensured that the various factors influencing milk production were properly accounted for, providing a robust and accurate estimation of the 270DMY. The adjusted 270DMY phenotype contains variance arising from additive and non-additive genetic sources as well as permanent environmental effects.

#### Estimating genetic variance and EBV of the 270-Day milk yield

For genetic parameter estimation and EBV, a simple linear mixed model was applied as follows:

y=Wa+e

where y represents the vector of 270DMY observations for individual buffaloes. W is the design matrix linking random additive animal effects to the observations. a denotes the vector of random additive genetic effects (animal effects), and e is the residual error term. The a were assumed to follow a multivariate normal distribution $u∼N(0,\;A^{-1}\sigma_{a}^{2})$, where $\sigma_{a}^{2}$ denotes the additive genetic variance component, and A is the additive relationship matrix constructed based on pedigree information. e was assumed to be independent and identically distributed following $e∼N(0,\;I\sigma_{e}^{2})$, where $\sigma_{e}^{2}$ is the residual variance component and I is the identity matrix.

The BGLR package in R was employed for statistical estimation of variance components and the posterior distributions of breeding values (Pérez & de los Campos, 2014). The model parameters were sampled from posterior distributions using a Gibbs sampling-based Markov Chain Monte Carlo (MCMC) algorithm. The analysis utilized 140,000 MCMC iterations, with the first 40,000 iterations discarded as burn-in. A thinning interval of 20 was applied, retaining every 20th sample for posterior analysis. Convergence diagnostics were evaluated to verify the adequacy of the MCMC sampling process using the coda package (Plummer et al., 2006).

The accuracy of the estimated breeding values (EBVs) was calculated using the following formula:

$$Accuracy_{i}=\sqrt{1-\frac{SD_{i}^{2}}{\sigma_{a}^{2}}}$$

In this formula, $SD_{i}$ represents the standard deviation of the posterior distribution of the genetic effects obtained from the Bayesian model fitting for the *i*th buffalo (Mrode, 2014).

## Result

### Test-day milk yield

**Fig. 1:**
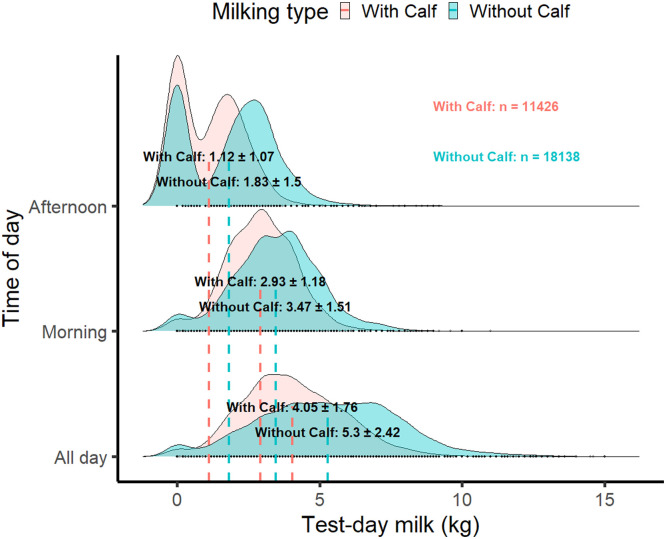
Test-day milk yield, unadjusted for possible effects, categorized by morning, afternoon, and the entire day. The plot displays the mean ± standard deviation for each group, further distinguished by milking type (with calf vs. without calf).

Fig. 1 presents the density distribution of TDM recorded during morning, afternoon, and across the entire day. The results indicate that milk yield varies substantially throughout the day, with the highest and most consistent production observed in the morning. The average daily milk yield in this study was 4.82 kg, with a maximum observed value of 15 kg. Notably, a pronounced peak at 0 kg in the afternoon distribution highlights a considerable number of records with no milk production during afternoon milking sessions.

The interaction plot (Supplementary file, Figure S1) clearly demonstrates the relationship between DM and the residuals of TDM, with separate trends for cows that were milked with and without their calves. The residuals of daily milk yield for buffaloes milked with their calves showed a slower increase in TDM. This is mainly due to the calves consuming milk during the milk letdown stimulation. Since records were collected daily at milking, it was not possible to hand-feed the milk, as the calves were accustomed to suckling from their mothers. The peak TDM was achieved around 75 days after calving. Conversely, buffaloes milked without their calves exhibited a different trend in TDM, with the peak achieved around 50 days after calving. After reaching the peak of production, the reduction in TDM was faster in the without-calf group, indicating that the presence of a calf probably contributes to longer milk production in buffaloes. Additionally, in the group milked without calves, the total daily milk (TDM) declined more gradually between approximately 150 and 250 days of lactation, followed by a steeper decrease after around the 250th day.

### Days open prediction

The regression analysis for predicting the DP revealed several significant factors (supplementary file, Table S2). The intercept was estimated at 209.9 ± 16.88 days. Among the MT, the 'Without Calf' category demonstrated a significant decrease on DP, with an estimate of −81.85 ± 19.38. SN also played a notable role. The spring season showed a positive association with DP compared to fall, while summer and winter seasons did not have statistically significant effects. Additionally, the AC was found to have a significant negative influence on DP, with an estimate of −7.543 ± 1.895.

### Fixed effects

The results of the identity-structured random animal linear mixed model analysis are summarized in supplementary file, Table S3. The findings reveal significant effects of the DM and its polynomial terms on performance parameters. The interaction between DM and the absence of a calf (MT: without calf) was also highly significant. Furthermore, the estimated increase in TDM by 0.3 kg (± 0.3) for the MT: without calf group was significant, suggesting that this amount represents the average milk consumption by calves in the groups where calves are present. Other covariates such as WC, AC, and their polynomial terms were found to be significant contributors to the model. The effect of AT on TDM was also highly significant (p-value < 0.001). The estimate of −0.012 (± 0.001) indicates that for each unit increase in average temperature, there is a decrease of 0.012 kg in the total TDM. This suggests that higher temperatures negatively affect TDM, highlighting the importance of considering environmental factors in the analysis.

Table 1 presents model-adjusted descriptive statistics for TDM: adjusted means for categorical factors (MT, CN, and SN) with pairwise contrasts (Δ), standard error, and 95 % confidence intervals, and average marginal effects for continuous covariates (e.g., AC, WC, AT, DM, and PD) expressed as kg per unit increase. Consistent with the fixed-effects results above, the marginal means show a curvilinear pattern for CN, where adjusted means peak at CN 3 (5.156 kg) and decline thereafter, lowest at CN 7 (2.713 kg). MT means are similar (with-calf 4.168 kg, without-calf 4.227 kg), consistent with the fixed-effect contrast of +0.30 kg/day for “without calf”. By SN, winter is highest and spring lowest. For continuous covariates, AC shows a positive slope (+0.262 kg/year); DM declines more slowly with calf (−0.012 kg/day) than without calf (−0.033 kg/day); PD is negative (−0.0054 kg/day); AT is adverse (−0.012 kg/ °C); and WC is positive (+0.0055 kg/kg).

**Table 1:** Model-adjusted descriptive statistics for test-day milk yield: adjusted means for categorical factors and average marginal effects for continuous covariates, each reported with standard errors (SE) and 95 % confidence intervals (CI); continuous effects are in kg per unit increase, and adjusted means are in kg per milking.

Effect	Type	Level	Estimate ± SE	95 % CI (lower to upper)
CN	Marginal mean (kg)	1	4.171 ± 0.328	3.527 to 4.814
		2	4.76 ± 0.216	4.336 to 5.184
		3	5.156 ± 0.15	4.863 to 5.45
		4	4.678 ± 0.158	4.368 to 4.988
		5	3.943 ± 0.226	3.5 to 4.385
		6	3.96 ± 0.311	3.35 to 4.57
		7	2.713 ± 0.393	1.944 to 3.483
MT	Marginal mean (kg)	With Calf	4.168 ± 0.324	3.534 to 4.802
		Without Calf	4.227 ± 0.321	3.598 to 4.855
SN	Marginal mean (kg)	Fall	4.193 ± 0.328	3.55 to 4.837
		Spring	3.867 ± 0.321	3.238 to 4.495
		Summer	4.23 ± 0.321	3.601 to 4.86
		Winter	4.499 ± 0.328	3.856 to 5.142
AC	Slope (kg/year)	Overall	0.262 ± 0.095	0.076 to 0.448
DM (by MT)	Slope (kg/day)	With Calf	−0.012 ± 0.0006	−0.013 to −0.011
		Without Calf	−0.033 ± 0.0005	−0.034 to −0.032
PD	Slope (kg/day)	Overall	−0.0054 ± 0.0004	−0.0062 to −0.0047
AT	Slope (kg/ °C)	Overall	−0.012 ± 0.001	−0.014 to −0.01
WC	Slope (kg/kg)	Overall	0.0055 ± 0.0005	0.0045 to 0.0065

### The 270-day milk yield

**Fig. 2:**
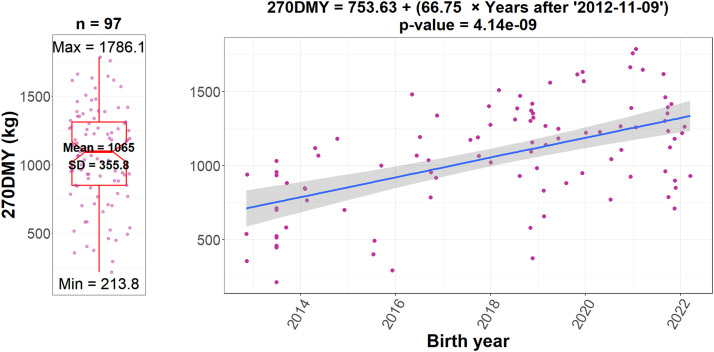
Distribution and Trend of 270-Day Milk Yield (270DMY). The left plot shows the distribution of 270DMY, and the right plot illustrates the trend of 270DMY over different year of birth, indicating a significant positive trend of phenotype performance over time for the breeding herd of Khuzestani buffalo maintained at the Department of Animal Science, Safiabad-Dezful Agricultural and Natural Resources Research and Education Center.

The estimated 270DMY using a mixed model is presented in Fig. 2. Our analysis revealed that the average 270DMY, with fixed values of AT = 26, MT = without calf, AC = 6, WC = 517, SN = summer, and CN = 3 was 1065 ± 355.8 kg. Additionally, the trend analysis of 270DMY over different years indicated a significant positive increase of 66.75 kg per year.

#### Heritability and EBV of the 270-Day milk yield

The 270DMY revealed significant insights into the variance components and genetic parameters of the Khuzestani buffalo population. Utilizing the BGLR package in R and a Gibbs sampling-based Markov Chain Monte Carlo (MCMC) approach, the posterior estimates of the model parameters were obtained (Table 2). The MCMC trace plot for the genetic variance confirmed good chain mixing and convergence, supporting the reliability of the posterior estimates (Supplementary Figure S2). The estimated heritability (h²) of the 270DMY was 0.433 with a standard deviation of 0.1228, reflecting a moderate genetic influence on this trait.

**Table 2:** Posterior estimates of variance components and model parameters for 270-day milk yield.

Parameter	Value
Log-likelihood At Posterior Mean	−665.82
Posterior mean of the log-likelihood	−683.59
Effective number of parameters	35.53
Deviance Information Criteria	1402.7
Additive genetic variance ($\sigma_{a}^{2}$)	62,532.6 (SD: 22,291.8; 95 % CrI: 28,920.5–115,357.9)
Residual variance ($\sigma_{e}^{2}$)	80,471 (SD: 19,383.5; 95 % CrI: 45,999.5–121,262.1)
Heritability (SD)	0.433 (SD: 0.1228; 95 % CrI: 0.215–0.688)

**Fig. 3:**
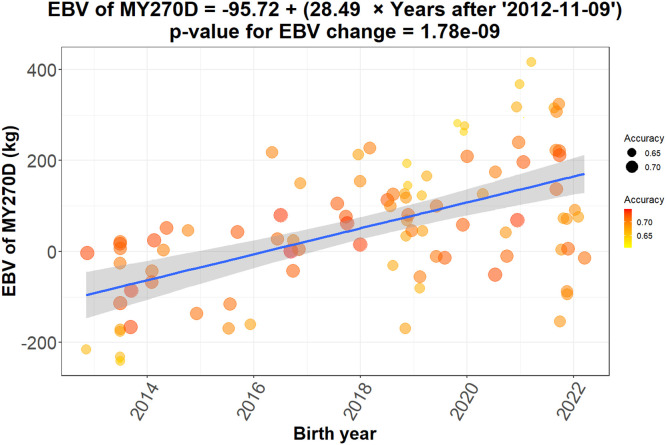
The plot illustrates the trend of estimated breeding values (EBVs) for the 270-Day Milk Yield (270DMY) in Khuzestani buffalo across different birth years. Each data point represents the EBV for a buffalo, with the x-axis showing the birth year and the y-axis indicating the EBV in kilograms. The color and size of the data points reflect the accuracy of the EBV estimates—points with higher accuracy are larger and displayed in darker red, while points with lower accuracy are smaller and closer to yellow. A fitted regression line (blue line) captures the overall trend, showing a statistically significant positive increase in EBVs over time (p-value < 0.001).

The analysis of EBV for the 270DMY across different birth years in Khuzestani buffalo demonstrated a consistent positive trend (Fig. 3). The regression analysis revealed an annual increase of approximately 28.5 kg in EBV, reflecting significant genetic progress for this economically important trait (p-value < 0.01). This improvement is attributed to the implementation of selective breeding programs targeting higher milk yield. These findings confirm that Khuzestani buffalo respond favorably to selection for milk yield under intensive management, supporting the feasibility of structured selection programs. This outcome provides a foundation for incorporating additional economic traits in subsequent multi-trait evaluations.

## Discussion

This study represents the first comprehensive evaluation of milk production performance and its genetic parameters in the Khuzestani buffalo under intensive management conditions. By analyzing detailed TDM records alongside management and environmental factors, we provide critical insights into both phenotypic and genetic variability in this important indigenous breed. Given the Khuzestani buffalo’s strategic role in Iran’s dairy sector and its adaptation to the region’s challenging climate, understanding its production potential and the underlying genetics is essential for effective breeding and management strategies.

Milk production in buffalo varies considerably across breeds and regions worldwide, reflecting differences in genetics, environment, and management. In Italy, where the Mediterranean buffalo is reared mainly for mozzarella cheese production, average lactation milk yield has been reported at around 2350 kg, with particularly high milk solids content (Catillo et al., 2002). In South Asia, where buffalo contribute significantly to the dairy economy, performance varies across breeds. In India, Murrah buffalo maintained in organized herds typically yield between 1700 and 1900 kg in 305-day lactations (Sahoo et al., 2014; Sigdel et al., 2015; Thiruvenkadan et al., 2014), although broader reviews place the breed’s yield range between 1365 and 2500 kg (Jamuna & Chakravarty, 2016; Kumar et al., 2022, 2017). In neighboring Pakistan, the Nili-Ravi breed demonstrates comparable performance, with average lactation yields ranging from 1700 to over 2100 kg, depending on herd and management conditions (Afzal et al., 2007; Cady et al., 1983; Chaudhry, 1992). Egyptian buffalo, which are an important dairy resource in North Africa, show mean lactation yields between 1175 and 1700 kg, with notable regional variation (Ahmed et al., 2024; El-Bramony, 2011). In Brazil, Murrah buffalo and their crosses produce a broad range of 1250–1800 L per lactation under different farm conditions, with controlled herd studies reporting means of about 1594 ± 590 kg in a 271-day lactation (Aspilcueta-Borquis et al., 2010b; Seno et al., 2010; Tonhati et al., 2000). In Turkey, where the Anatolian buffalo is kept largely in smallholder systems, yields remain comparatively lower, typically ranging from 800 to 1600 kg per lactation (Yilmaz et al., 2012). Moreover, previous studies shine a light on the productivity of the Iranian Khuzestani buffalo, a breed often overlooked in global comparisons. Madad et al. (2013a) reported an average TDM of 8.96 ± 2.69 kg in Khuzestani buffaloes, suggesting a relatively high production potential for this breed. In contrast, our findings from an intensively managed herd showed a markedly lower average of 4.82 ± 2.27 kg. One important consideration is that Madad et al. (2013a) calculated TDM based on a 240-day period, which likely contributed to the higher average yield compared to our results, as shorter reference periods often capture peak production more strongly than extended lactation records. Supporting this interpretation, analyses of the same data set have reported average lactation yields of 2303.6 kg in the second lactation (Madad et al., 2013b) and an overall mean of 2285.08 kg (Baharizadeh, 2012), both of which are considerably higher than our calculated herd average of 1065 kg for the thrid calving. Given that our herd was kept under relatively favorable conditions for an intensive system, this discrepancy is unlikely to be explained solely by environmental differences. A more plausible explanation lies in the recording methodology. It is possible that previous studies may have inadvertently overestimated production by preferentially recording milk yields from the highest-performing animals or from selected farms with better resources. Because our herd was established by collecting buffalo from across the Khuzestan province, it represents a broad and realistic cross-section of the population, making it unlikely that genetic differences alone account for such large discrepancies. Rather, these contrasts underscore the impact of data collection approaches, sampling strategies, lactation length used for averaging, and possible selection bias in shaping reported estimates of buffalo productivity. Such methodological considerations are critical when interpreting milk yield data and should be carefully accounted for before making generalizations about the genetic potential of Khuzestani buffaloes.

The reproductive capacity of a herd is a key determinant of profitability in buffalo farming, and poor reproductive performance remains one of the most common reasons for culling in dairy buffalo herds (Kim & Jeong, 2018; Shao et al., 2021). Among the various indicators of reproductive efficiency, DO is considered one of the most important traits, as it directly reflects the interval between calving and successful conception. Environmental factors significantly influence reproductive performance in buffaloes. Seasonal variations, particularly temperature extremes, can disrupt estrous cycles and extend DO, leading to longer calving intervals and reduced productivity (Mohamed et al., 2024; Shah et al., 1989; Vale, 2007; Zicarelli, 2017). Studies on Egyptian buffaloes have demonstrated that factors like age at first calving, parity, and calving season notably impact DO, underscoring the importance of optimizing these variables to enhance reproductive efficiency (Mohamed et al., 2024). It has been shown that calf suckling significantly improves reproductive performance in buffaloes, as increased mother–calf contact shortens the postpartum ovulation interval and estrus period while enhancing milk production (Qureshi & Ahmad, 2008). Suckling exerts a profound influence on postpartum reproductive performance in buffaloes, often delaying the onset of ovarian cyclicity and estrus. Studies have shown that buffaloes nursing their calves experienced longer intervals to first ovulation and estrus compared to weaned animals, with primiparous buffaloes being particularly affected (El-Fouly et al., 1976; Qureshi & Ahmad, 2008; Rijasnaz et al., 2014). Our findings indicate that the average days open (DO) in this population was 209.9 ± 16.88 days, notably higher than previously reported values for this breed, where a calving interval of 400.6 ± 75.5 days corresponds to an estimated DO of approximately 90 days, assuming an average gestation length of 310 days (Morammazi et al., 2007). Importantly, our study demonstrates for the first time in this breed that factors such as season, age, and calf suckling can exert a significant influence on reproductive performance by modulating the calving interval. These results highlight the complex interplay of biological and management factors in shaping fertility outcomes in buffalo herds. For example, milking mothers without their calves was associated with a reduction in calving interval by 81.85 days, a substantial effect that can markedly enhance reproductive efficiency and, consequently, improve herd profitability.

Buffalo show strong dam–calf bonding that can facilitate milk let-down; however, under mechanical milking or when milking routines or feed provision are suboptimal, this calf-dependent response may be reduced. Consequently, separation of the dam from her calf is often more stressful for both mother and offspring compared to taurine cattle (Kamboj & Kumar, 2013). However, Bharti et al. (2015) reported that weaning did not adversely affect the health or immunity of buffalo calves compared to those allowed to suckle naturally. Despite these insights, research on the impact of weaning versus suckling on the milk production of buffalo dams remains limited. For instance, one study found that average daily milk yield was significantly lower in weaned buffaloes compared to suckled animals (6.31 vs. 8.99 kg/day, *P* < 0.01). In contrast, our results indicate that, on average, buffaloes milked without calf suckling produced 0.3 ± 0.3 kg more milk per day, likely due to the milk normally consumed by the calf during milking. Furthermore, for the first time, we demonstrate that milking type (MT) can influence the lactation curve in buffaloes, as the interaction between daily milk (DM) and absence of the calf (MT: Without Calf) was highly significant. The results show distinct lactation patterns for buffaloes milked with or without their calves. Buffaloes milked with calves reached peak daily milk yield later and maintained production longer, likely because calves consume milk during letdown, prolonging lactation, whereas those milked without calves peaked earlier but declined faster. Pregnancy is also known to reduce milk yield in dairy buffaloes, particularly after the 8th week post-conception (Khan et al., 2011; Qureshi, 2009). Interestingly, our results show a slower decline in milk yield after approximately the 150th day of milking, which corresponds to roughly the 8th week post-conception. This slower decrease aligns with the physiological adaptations occurring in the dam during the first weeks of gestation, when the maternal body is still adjusting to the developing fetus (Fiore et al., 2018). Although hormonal and metabolic shifts can reduce milk yield, these changes appear to progress gradually, allowing buffaloes to maintain relatively stable milk production for a longer period. This finding suggests that early pregnancy may have a milder impact on lactation than previously assumed, highlighting the resilience of buffalo dams in balancing fetal development with ongoing milk synthesis. However, the decline in milk yield became more pronounced after approximately the 240th day of milking in the groups without calves, corresponding to around the 15th week of pregnancy on average. This aligns with previous findings indicating that the impact of pregnancy on milk production generally becomes significant from the third month of gestation onward, as the dam’s physiological priorities shift toward supporting fetal growth (Olori et al., 1997). This discrepancy highlights the potential for strategic calf management during milking to optimize milk production while maintaining calf welfare, ultimately improving overall herd productivity and profitability.

Our analysis of fixed effects revealed that, beyond calf presence, several management and environmental factors significantly shaped milk production in Khuzestani buffaloes. TDM yield followed a clear nonlinear trajectory across days in milk, confirming the characteristic lactation curve of buffaloes reported previously (Abdel-Salam et al., 2011; Macciotta et al., 2006). Age at calving exerted a strong quadratic effect, with younger and older animals producing less milk compared to those calving at intermediate ages, consistent with findings in other dairy buffalo populations (Catillo et al., 2002). Similarly, parity influenced milk yield, with animals in their second and third calving performing best, while yields declined in higher parities, likely reflecting cumulative physiological stress and advancing reproductive age (Pawar et al., 2012). Seasonal influences were also evident: yields were depressed in spring and enhanced in winter, a trend aligning with the well-documented sensitivity of buffaloes to thermal stress and their improved performance in cooler climates (Catillo et al., 2002; Pawar et al., 2012). Temperature had a clear negative association with yield, further underscoring the vulnerability of buffaloes to heat stress compared to taurine cattle (Dash et al., 2016; Petrocchi Jasinski et al., 2023; Sinha & Minett, 1947). Collectively, these results emphasize that milk yield in buffaloes reflects a dynamic interaction between physiology, environment, and management. Importantly, they highlight the need for context-specific strategies—such as optimizing calving season, managing heat stress, and supporting higher-parity animals—to sustain productivity in buffalo herds under diverse production systems.

The heritability estimate of 0.433 for 270-day milk yield (270DMY) in Khuzestani buffalo indicates a moderate genetic influence, showing that selective breeding can effectively improve this trait. This value is consistent with estimates reported in other buffalo populations, generally ranging from 0.14 to 0.61 (Aspilcueta-Borquis et al., 2010a; Easa et al., 2022; Madad et al., 2013b; Rosati and Van Vleck, 2002) , and is slightly higher than estimates for Iranian buffaloes, often around 0.09–0.33 (Madad et al., 2013a, b; Baharizadeh, 2012). Beyond the genetic parameters, our results also revealed a clear phenotypic improvement, with an average increase of 66.75 kg in 270DMY across the study period. This trend complements the positive genetic trajectory, as reflected in an annual gain of about 28.5 kg/year in EBV, and together they provide strong evidence that selective breeding programs in Khuzestan are translating into measurable improvements at both the genetic and population levels. Comparable genetic progress has been reported in Nili-Ravi and Murrah buffaloes in South Asia (Ahmad, 2007; Bashir et al., 2025; Sharma et al., 2024), suggesting that Khuzestani buffaloes are keeping pace with or even exceeding gains achieved in other well-established breeding programs. The combination of moderate heritability, favorable phenotypic responses, and steady genetic improvement highlights both the potential and the ongoing success of genetic selection in this population. However, long-term gains will depend on maintaining balanced breeding goals that incorporate fertility and adaptability alongside yield, as emphasized in dairy cattle breeding (Cole et al., 2021). Overall, these findings demonstrate that Khuzestani buffaloes are genetically improving for milk yield at a rate consistent with or exceeding other Iranian and international populations, underscoring the importance of continued investment in structured recording and breeding programs to strengthen productivity and resilience.

A key limitation of this study is the relatively small number of animals (*n* = 97) available for genetic evaluation, which may reduce the robustness of variance-component and trend estimates. However, the large number of test-day records per animal and the high EBV accuracy help to mitigate this constraint and support the reliability of the estimated genetic parameters. Moreover, this report is a narrow focus on yield. Although our selection index already includes milk composition, composition records during this window are still maturing and not yet suitable for stable genetic parameterization. Accordingly, this study should be viewed as a first-phase genetic assessment, designed to quantify the heritable variation and response potential of the most economically important trait under a newly established intensive management system. In the next phase, we will deliver multi-trait evaluations (yield + composition), estimate genetic correlations, and update the selection index to better balance volume and quality. As composition, fertility, and adaptability data become robust, these traits will be integrated to develop balanced, economically optimized breeding programs for Khuzestani buffalo.

## Conclusion

This study provides the first population-level genetic baseline for Khuzestani buffalo under intensive farming: moderate heritability of 270-day milk yield, a positive EBV trend (∼+2.7 %/year), and a phenotypic gain of ∼+6.3 % over the study period. Yield peaked at CN=3, improved with greater AC, declined with DM (shallower with calf), was highest in winter, and decreased with AT. These results indicate that selection on yield is currently effective, and that targeted management—milking routine/calf management, calving age/parity planning, and heat mitigation—can further enhance realized gains. Looking ahead, these results provide a practical foundation for balanced, multi-trait breeding programs that pair milk yield with fertility, adaptability, and heat tolerance to secure sustainable gains under changing conditions. Prioritizing systematic phenotypic/genetic recording will enable precision, index-based selection and continuous refinement of herd performance, positioning Khuzestani buffalo as a model for conserving and improving indigenous dairy breeds.

## Funding

This work was supported by institutional resources from the Agricultural Research, Education & Extension Organization (AREEO) as part of the establishment of the first nucleus breeding program for Khuzestani buffalo at the Department of Animal Science, Safiabad-Dezful Agricultural and Natural Resources Research and Education Center, Dezful, Iran. No external funding was received for the design of the study, data collection, analysis, interpretation, or preparation of the manuscript.

## Ethical statement

The authors affirm that this study adheres to the ethical standards outlined by Elsevier’s publishing policies. All data were collected and analyzed with integrity, transparency, and scientific rigor. The manuscript presents original work that has not been published elsewhere and is not under consideration by any other journal. Proper acknowledgment has been given to all sources and contributors. No part of the study involves human participants or animal experimentation. All authors have reviewed and approved the final version of the manuscript and agree to be accountable for all aspects of the work, ensuring that questions related to its accuracy or integrity are appropriately investigated and resolved.

## CRediT authorship contribution statement

**Kobra Karimi:** Writing – original draft, Investigation, Formal analysis. **Mohammad Taghi Beigi Nassiri:** Writing – review & editing, Validation, Supervision, Conceptualization. **Mahmoud Amiri Roudbar:** Writing – review & editing, Visualization, Validation, Supervision, Project administration, Methodology, Data curation, Conceptualization. **Alireza Jolazadeh:** Writing – review & editing, Methodology, Investigation.

## Declaration of competing interest

The authors declare that they have no known competing financial interests or personal relationships that could have appeared to influence the work reported in this paper.

## Appendix Supplementary materials

Image, application 1
