# Supplementary material for: Genetic and phenotypic evaluation of milk production in Khuzestani buffalo under intensive farming conditions
Source: Vet Anim Sci. 2026 Jan 30;31:100589. doi: 10.1016/j.vas.2026.100589 (PMC12906111; doi:10.1016/j.vas.2026.100589)
Supplement: Supplementary file 1 [file mmc1.docx]

**Genetic and phenotypic evaluation of milk production in Khuzestani buffalo under intensive farming conditions**

Kobra Karimi, Mohammad Taghi Beigi Nassiri, Mahmoud Amiri Roudbar, Alireza Jolazadeh

**Table S1.** Pedigree structure and inbreeding statistics for the Khuzestani buffalo population used in this study.

| **Parameter** | **Value** |
| --- | --- |
| Number of individuals | 1125 |
| Number of sire | 58 |
| Number of dam | 368 |
| Number of inbreds | 63 |
| Number of individuals with both known parents | 696 |
| Number of individuals with no progeny | 699 |
| Average family size | 2.24 |
| Average inbreeding coefficients | 0.0075 |
| Average inbreeding coefficients in the inbreds | 0.1344 |
| Maximum of inbreeding coefficients | 0.25 |
| Average numerator relationships | 0.018 |
| Average pedigree depth (maximum generation) | 1.56 (5) |

**Table S2.** Regression analysis results for the best model fitting of days open in buffalo.

| **Effect** | **Estimate** | **Std. Error** | **t value** | **P-value** |
| --- | --- | --- | --- | --- |
| Intercept | 209.9 | 16.88 | 12.44 | 5.23e-30 |
| MT: Without Calf | -81.85 | 19.38 | -4.224 | 3.01e-05 |
| SN: Spring | 69.68 | 17.37 | 4.011 | 7.29e-05 |
| SN: Summer | -7.003 | 15.94 | -0.439 | 0.661 |
| SN: Winter | -5.715 | 15.94 | -0.359 | 0.720 |
| AC | -7.543 | 1.895 | -3.979 | 8.28e-05 |

**Table S3.** Effects of management and environmental factors on test-day milk yield in Khuzestanian buffalo.

| **Effect^1^** | **Estimate ± SE** | **P-value** |
| --- | --- | --- |
| (Intercept) | 838.13 ± 44.80 | <1.00E-16 |
| DM | 0.14 ± 0.05 | 5.69E-03 |
| DM^2^ | -3.50e-03 ± 2.96e-03 | 0.24 |
| DM^3^ | 4.19e-05 ± 8.58e-05 | 0.63 |
| DM^4^ | -8.32e-08 ± 1.39e-06 | 0.95 |
| DM^5^ | -3.23e-09 ± 1.33e-08 | 0.81 |
| DM^6^ | 3.56e-11 ± 7.82e-11 | 0.65 |
| DM^7^ | -1.65e-13 ± 2.74e-13 | 0.55 |
| DM^8^ | 3.67e-16 ± 5.25e-16 | 0.48 |
| DM^9^ | -3.24e-19 ± 4.24e-19 | 0.45 |
| MT: Without Calf | 0.30 ± 0.30 | 0.32 |
| WC | -8.36 ± 0.43 | <1.00E-16 |
| WC^2^ | 0.03 ± 1.63e-03 | <1.00E-16 |
| WC^3^ | -6.43e-05 ± 3.05e-06 | <1.00E-16 |
| WC^4^ | 6.12e-08 ± 2.81e-09 | <1.00E-16 |
| WC^5^ | -2.29e-11 ± 1.02e-12 | <1.00E-16 |
| AC | -8.63 ± 2.80 | 2.02E-03 |
| AC^2^ | 1.72 ± 0.84 | 0.04 |
| AC^3^ | -0.14 ± 0.12 | 0.24 |
| AC^4^ | 4.95e-03 ± 8.28e-03 | 0.55 |
| AC^5^ | -4.37e-05 ± 2.21e-04 | 0.84 |
| SN: Spring | -0.56 ± 0.05 | <1.00E-16 |
| SN: Summer | -0.49 ± 0.06 | <1.00E-16 |
| SN: Winter | -0.08 ± 0.05 | 0.09 |
| CN: 2 | 0.09 ± 0.15 | 0.58 |
| CN: 3 | -0.26 ± 0.29 | 0.37 |
| CN: 4 | -1.23 ± 0.40 | 2.17E-03 |
| CN: 5 | -2.12 ± 0.49 | 1.31E-05 |
| CN: 6 | -2.22 ± 0.56 | 7.76E-05 |
| CN: 7 | -4.02 ± 0.64 | 2.97E-10 |
| DP | -5.27e-03 ± 3.99e-04 | <1.00E-16 |
| AT | -24.29 ± 1.84 | <1.00E-16 |
| AT^2^ | -8.77 ± 1.52 | 8.07E-09 |
| DM*MT_Without calf_ | 0.33 ± 0.05 | 6.54E-10 |
| DM^2^*MT_Without calf_ | -0.02 ± 3.25e-03 | 6.72E-10 |
| DM^3^*MT_Without calf_ | 5.81e-04 ± 9.67e-05 | 1.94E-09 |
| DM^4^*MT_Without calf_ | -9.49e-06 ± 1.60e-06 | 3.12E-09 |
| DM^5^*MT_Without calf_ | 9.23e-08 ± 1.58e-08 | 4.77E-09 |
| DM^6^*MT_Without calf_ | -5.45e-10 ± 9.45e-11 | 8.14E-09 |
| DM^7^*MT_Without calf_ | 1.91e-12 ± 3.37e-13 | 1.55E-08 |
| DM^8^*MT_Without calf_ | -3.65e-15 ± 6.60e-16 | 3.16E-08 |
| DM^9^*MT_Without calf_ | 2.93e-18 ± 5.43e-19 | 6.68E-08 |

^1^DM; day of milking (1–270 days), CN; calving number (1–7), AC; age at calving (2.7–12 years), MT; milking type (with or without calf presence for milk letdown), WC; weight at calving (346–764 kg), AT; average temperature on the milking day (8.5–43.1 °C), DP; days pregnant (0–244 days), and SN; season of the year (spring, summer, fall, or winter).


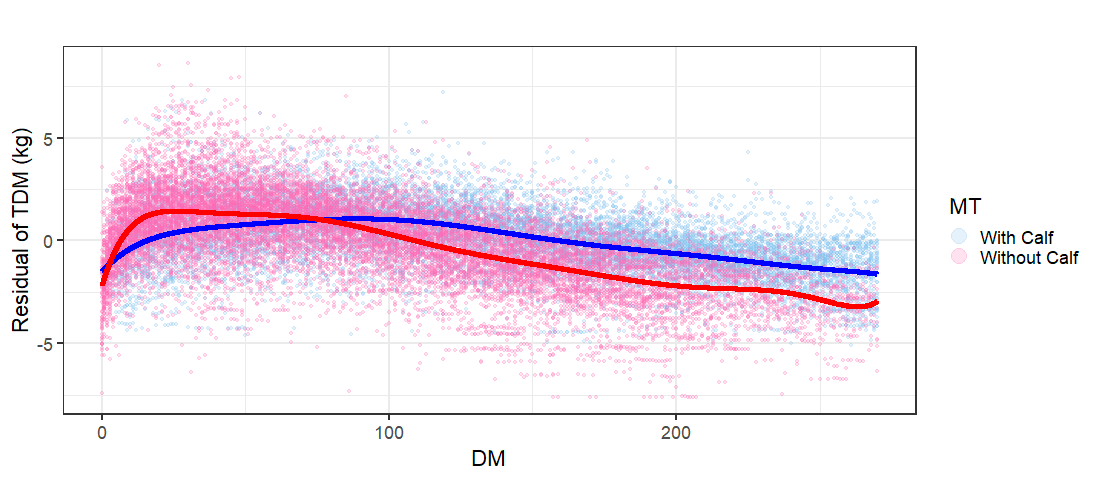
**Figure S1**. The plot shows the residual of test-day milk (TDM) in kg against day of milking (DM) for two groups of milking type (MT): those milked with a calf (blue) and those milked without a calf (red). Data points are scattered, with blue and pink dots representing individual measurements for buffaloes with and without a calf, respectively. The lines (by fitting separate polynomial models with 9th degree) represent the trend for each group, showing how the residual milk yield changes over time.


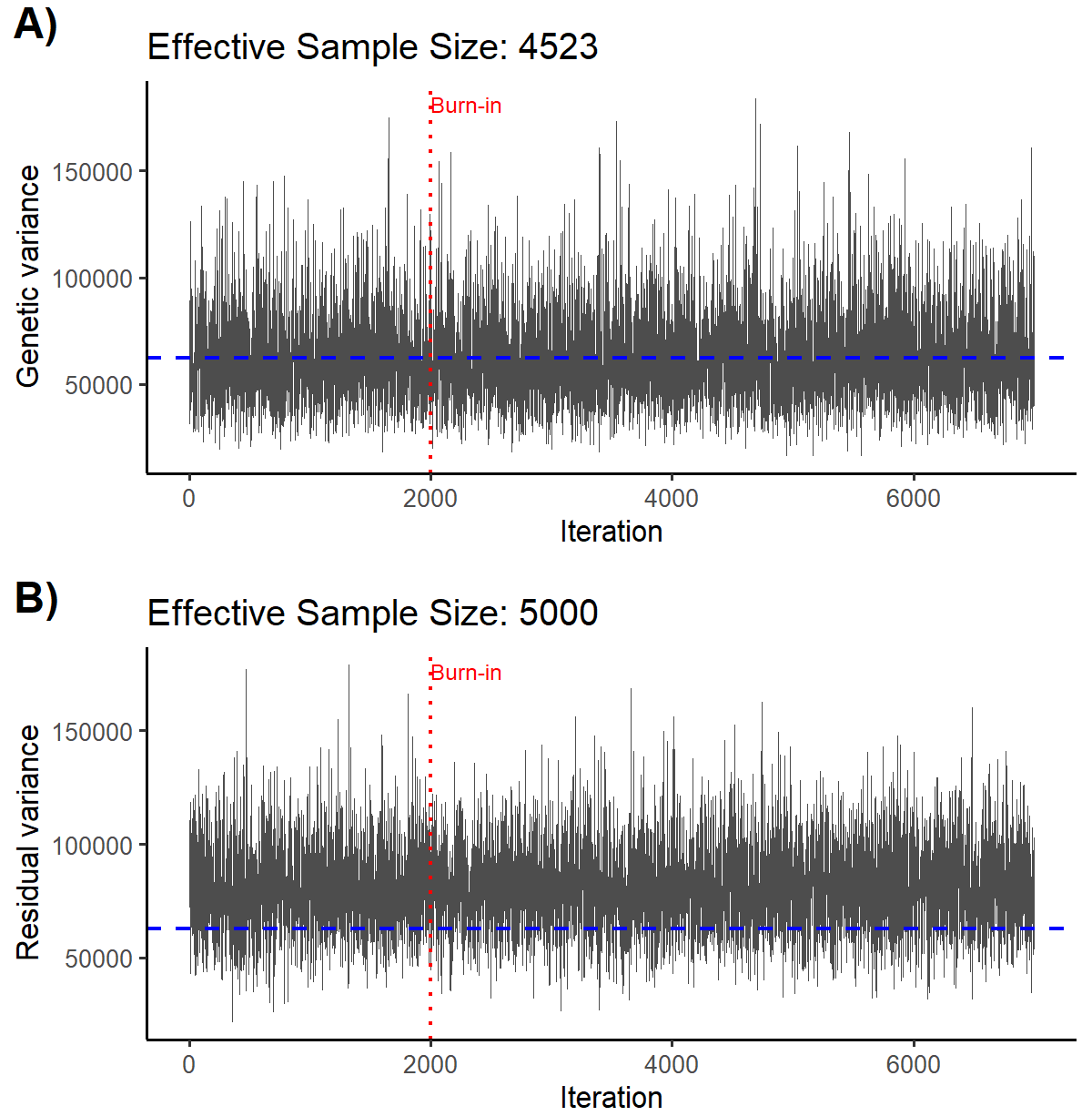


**Figure S2.** Trace plots of the MCMC samples for the (A) additive genetic variance and (B) residual variance. Both traces show stable mixing with no apparent trends following burn-in, indicating satisfactory convergence. The effective sample sizes for both parameters were close to 5,000 post–burn-in samples, reflecting low autocorrelation and good sampling efficiency. The red dotted vertical line denotes the burn-in point, and the blue dotted horizontal line indicates the posterior mean of the respective variance component.
